# Relationship between oral hygiene knowledge, source of oral hygiene knowledge and oral hygiene behavior in Japanese university students: A prospective cohort study

**DOI:** 10.1371/journal.pone.0236259

**Published:** 2020-07-23

**Authors:** Daiki Fukuhara, Daisuke Ekuni, Kota Kataoka, Ayano Taniguchi-Tabata, Yoko Uchida-Fukuhara, Naoki Toyama, Toshiki Yoneda, Yoshio Sugiura, Md. Monirul Islam, Hikari Saho, Yoshiaki Iwasaki, Manabu Morita

**Affiliations:** 1 Department of Preventive Dentistry, Okayama University Hospital, Okayama, Japan; 2 Department of Preventive Dentistry, Okayama University Graduate School of Medicine, Dentistry and Pharmaceutical Sciences, Okayama, Japan; 3 Department of Oral Morphology, Okayama University Graduate School of Medicine, Dentistry and Pharmaceutical Sciences, Okayama, Japan; 4 Advanced Research Center for Oral and Craniofacial Sciences, Okayama University Dental School, Okayama, Japan; 5 Health Service Center, Okayama University, Okayama, Japan; Manipal College of Dental Sciences, INDIA

## Abstract

The aim of this prospective cohort study was to examine whether oral hygiene knowledge, and the source of that knowledge, affect oral hygiene behavior in university students in Japan. An oral exam and questionnaire survey developed to evaluate oral hygiene knowledge, the source of that knowledge, and oral hygiene behavior, such as the frequency of tooth brushing and regular dental checkups and the use of dental floss, was conducted on university student volunteers. In total, 310 students with poor tooth brushing behavior (frequency of tooth brushing per day [≤ once]), 1,963 who did not use dental floss, and 1,882 who did not receive regular dental checkup during the past year were selected. Among these students, 50, 364, and 343 in each respective category were analyzed in over the 3-year study period (follow-up rates: 16.1%, 18.5%, and 18.2%, respectively). The odds ratios (ORs) and 95% confidence intervals (CIs) for oral hygiene behavior were calculated based on oral hygiene knowledge and the source of that knowledge using logistic regression models. The results showed that dental clinics were the most common (> 50%) source of oral hygiene knowledge, and that a more frequent use of dental floss was significantly associated with dental clinics being a source of oral hygiene knowledge (OR, 4.11; 95%CI, 1.871–9.029; *p* < 0.001). In addition, a significant association was seen between dental clinics being a source of oral hygiene knowledge and more frequent regular dental checkups (OR, 13.626; 95%CI, 5.971–31.095; *p* < 0.001). These findings suggest the existence of a relationship between dental clinics being the most common source of oral hygiene knowledge and improved oral hygiene behavior in Japanese university students.

## Introduction

Appropriate oral hygiene behavior, including frequent daily tooth brushing, using dental floss, and receiving regular dental checkups, can help prevent dental caries and periodontal disease [1–6]. Oral hygiene behavior is related to a variety of factors, including oral hygiene knowledge [7–9]. In Japan, previous studies reported that university students with better oral hygiene knowledge practiced better oral hygiene behavior [10, 11]. In other countries, similar results have been reported [12–14]. Furthermore, it has been reported that students who had acquired dental knowledge during the university life improved their oral health status [15].

Various sources of oral hygiene knowledge, including television [16–18], schools [19], and dental clinics [20, 21], have been reported to be associated with oral hygiene behavior. We previously conducted a cross-sectional study to investigate the associations between oral hygiene knowledge, the source of that knowledge, and oral hygiene behavior in a group of new university students [11]. The results suggested that having better oral hygiene knowledge, as well as having dental clinics as the most common source of oral hygiene knowledge, were associated with better oral hygiene behavior. Thus, when university students have oral hygiene knowledge from dental clinics, they may improve oral hygiene behavior. However, it remains unclear whether oral hygiene knowledge and the source of that knowledge affect oral hygiene behavior in university students in Japan, and there are little prospective cohort studies.

We hypothesized that both having oral hygiene knowledge and obtaining the knowledge form dental clinics improve oral hygiene behavior in university students in Japan. The purpose of the present prospective cohort study was to investigate the relationship between oral hygiene knowledge, the source of that knowledge, and improvement of oral hygiene behavior in university students in Japan.

## Materials and methods

### Sample size calculation

We estimated the sample size using G*Power version 3.1.9.6 statistical software. For chi-squared test, this software computed power for a test of the null hypothesis in which the event rate in the two groups was identical. According to a previous study [11], we calculated the effect size to be 0.205 and required the minimum sample size of 187 in groups to detect significant differences in the oral hygiene behaviors with 80% power and a two-sided significance level of 5%.

### Study population

Baseline data for use in this prospective cohort study were obtained from first-year Okayama university students (undergraduate students from all faculties) who had received general health and oral examinations at the Okayama University Health Service Center in April 2014. Japanese Okayama university students aged 18–24 years who fully completed a questionnaire and whose responses indicated poor oral hygiene behavior (frequency of tooth brushing per day ≤ once, no use of dental floss, and no regular dental visits) at baseline were included. All students volunteered to undergo general health and oral examinations for follow-up in April 2017, before graduation. Students who did not undergo an oral examination or provide complete questionnaire data at follow-up were excluded.

### Ethics procedures and informed consent

The study protocol was approved by the ethics committees of Okayama University Graduate School of Medicine, Dentistry and Pharmaceutical Sciences and Okayama University Hospital (No. 1060). The study was conducted and reported in accordance with the Strengthening the Reporting of Observational studies in Epidemiology (STROBE) guidelines. Informed verbal consent was obtained from all participants before the study began. The verbal consent was documented by signature in the questionnaire.

### Self-questionnaires

All students completed self-report questionnaires in Japanese language at both baseline (2014) and follow-up (2017).

### Oral hygiene knowledge

The students were asked whether they could explain a variety of dental terms (e.g., dental plaque, calculus, periodontal disease, temporomandibular disorder, dental floss, topical application of fluoride, fluoride-containing mouthwash, fissure sealant and 8020 movement (a social campaign in Japan aimed at promoting the retention of 20 or more of one’s own teeth at the age of 80 years) [10, 22] (S1 and S2 Tables).

### Source of oral hygiene knowledge

The students were also asked where they had acquired most of their oral hygiene knowledge (e.g., internet, television, dental clinics, family, school) (S1 and S2 Tables).

### Oral hygiene behavior

The students were also asked about their oral hygiene behavior, such as their frequency of tooth brushing per day (≥ twice/≤ once) and use of dental floss (yes/no), and whether they had received regular dental checkups during the past year (yes/no) [5, 6] (S1 and S2 Tables). Students who showed improved their oral hygiene behavior were defined as the improved group, and those who did not as the non-improved group.

### Oral examinations

Five dentists (D.E., T.A., S.M., M.Y-T., and K.K.) assessed the students’ periodontal status using the Community Periodontal Index (CPI) version 4 [23] using a CPI probe (YDM, Tokyo, Japan). Six sites (mesiobuccal, mid-buccal, distobuccal, distolingual, mid-lingual, and mesiolingual) were examined on each tooth. For the periodontal examinations, the following 10 teeth were selected: the upper right and lower left central incisors and two molars in each posterior sextant. Among these 10 teeth, the percentage exhibiting bleeding on probing (%BOP) was also assessed because compared with probing depth, BOP is an earlier and more sensitive indicator of gingival inflammation [10, 24]. The Oral Hygiene Index-Simplified (OHI-S) [25] was used to evaluate the students’ oral hygiene status. Calibration between five dentists was performed before the oral examination, and the intra- and inter-examiner reliabilities of the CPI scores as evaluated by κ statistics were both > 0.8 (the inter-examiner reliabilities of the CPI scores κ statistics: 0.815; the intra-examiner reliabilities of the CPI scores κ statistics: 0.868–1.000).

Furthermore, in each group, we investigated the relationship between oral hygiene behavior and increased BOP (worsened %BOP), periodontal pocket depth (PPD) (worsened PPD), and OHI-S scores (worsened OHI-S).

### Statistical analyses

Five dentists (A.T.T., N.T., Y.S., M.M.I, H.S.) collected the anonymous data. We used SPSS (version 25; IBM, Tokyo, Japan) for all statistical analyses, with *p* values < 0.05 considered to indicate statistical significance. Significant differences between the improved and non-improved oral hygiene behavior groups were determined using the fisher’s exact test and chi-square test.

Odds ratios (ORs) and 95% confidence intervals (CIs) were calculated using logistic regression model. The onset of each type of oral hygiene behavior (frequency of daily tooth brushing and using dental floss, and having regular dental visits) was used as the dependent variable, and in accordance with a previous study, oral hygiene knowledge, the source of that knowledge, sex, and age were used as independent variables associated with outcomes in a multiple logistic regression model. Backward elimination method was used to select the final model.

Significant differences between baseline and follow-up were investigated using the McNemar–Bowker test, paired *t* test, or Wilcoxon signed-rank test. Changes in periodontal (%BOP, PPD) and oral hygiene status (OHI-S) from baseline to follow-up were classified into two groups: i) worsened groups, and ii) stable groups. The relationship between oral hygiene behavior and the worsening in periodontal and oral hygiene status was investigated using the chi-squared test.

## Results

### Study population

**Fig 1:**
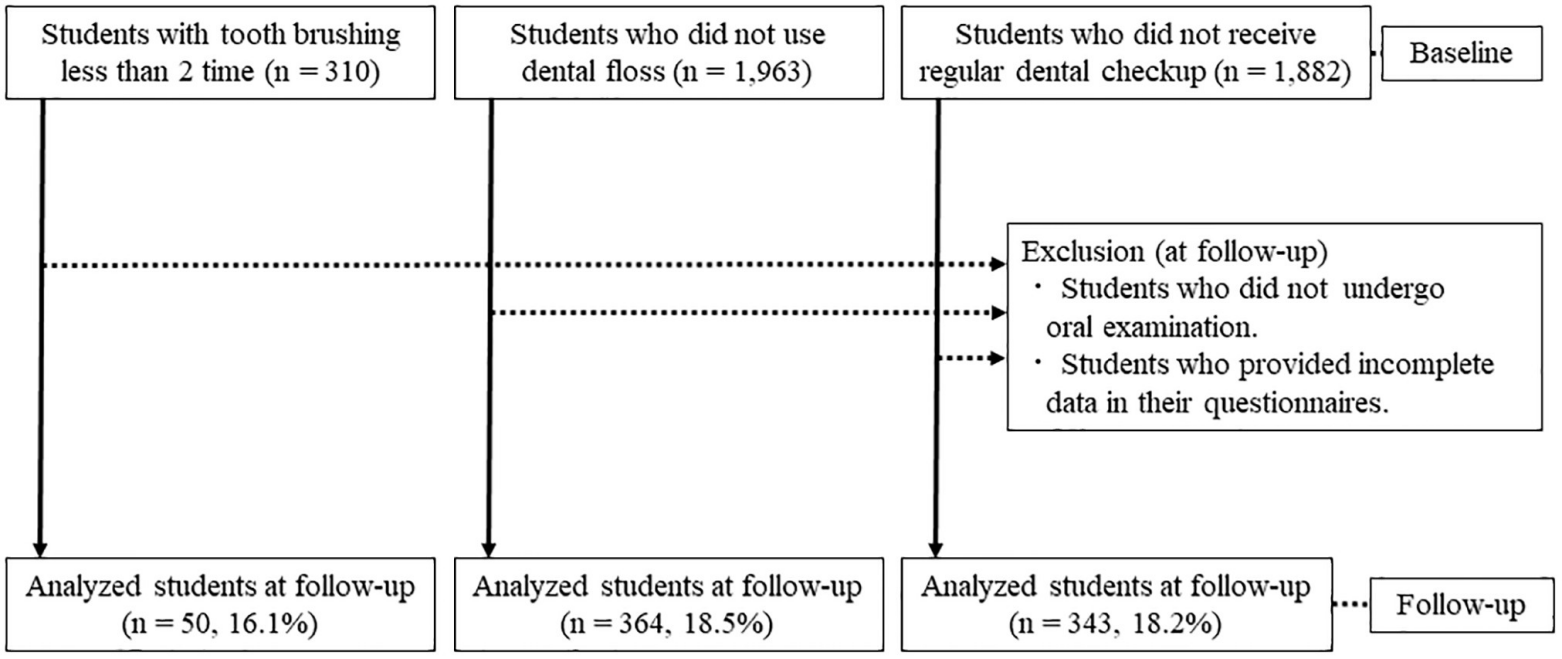
Flowchart. Students with poor oral hygiene behavior at baseline were classified into respective groups. Each group was analyzed at follow-up.

Baseline data (n = 2,220) were obtained from the oral examinations in April 2014. The students who met the inclusion criteria were classified into each poor oral hygiene behavior group as below. A flowchart of the participants in the present cohort study from baseline to follow-up is shown in Fig 1. Based on the baseline data, we selected 310 students with infrequent tooth brushing (frequency of tooth brushing per day [≤ once]), 1,963 who did not use dental floss, and 1,882 who did not have regular dental checkups for inclusion in the analysis. Of these students, 50, 364, and 343 in each respective category were analyzed (follow-up rates: 16.1%, 18.5%, and 18.2%, respectively). In addition, participants were examined for systemic disease. There were no systemic diseases and no significant differences in periodontal condition at baseline between the improved and non-improved groups.

**Fig 2:**
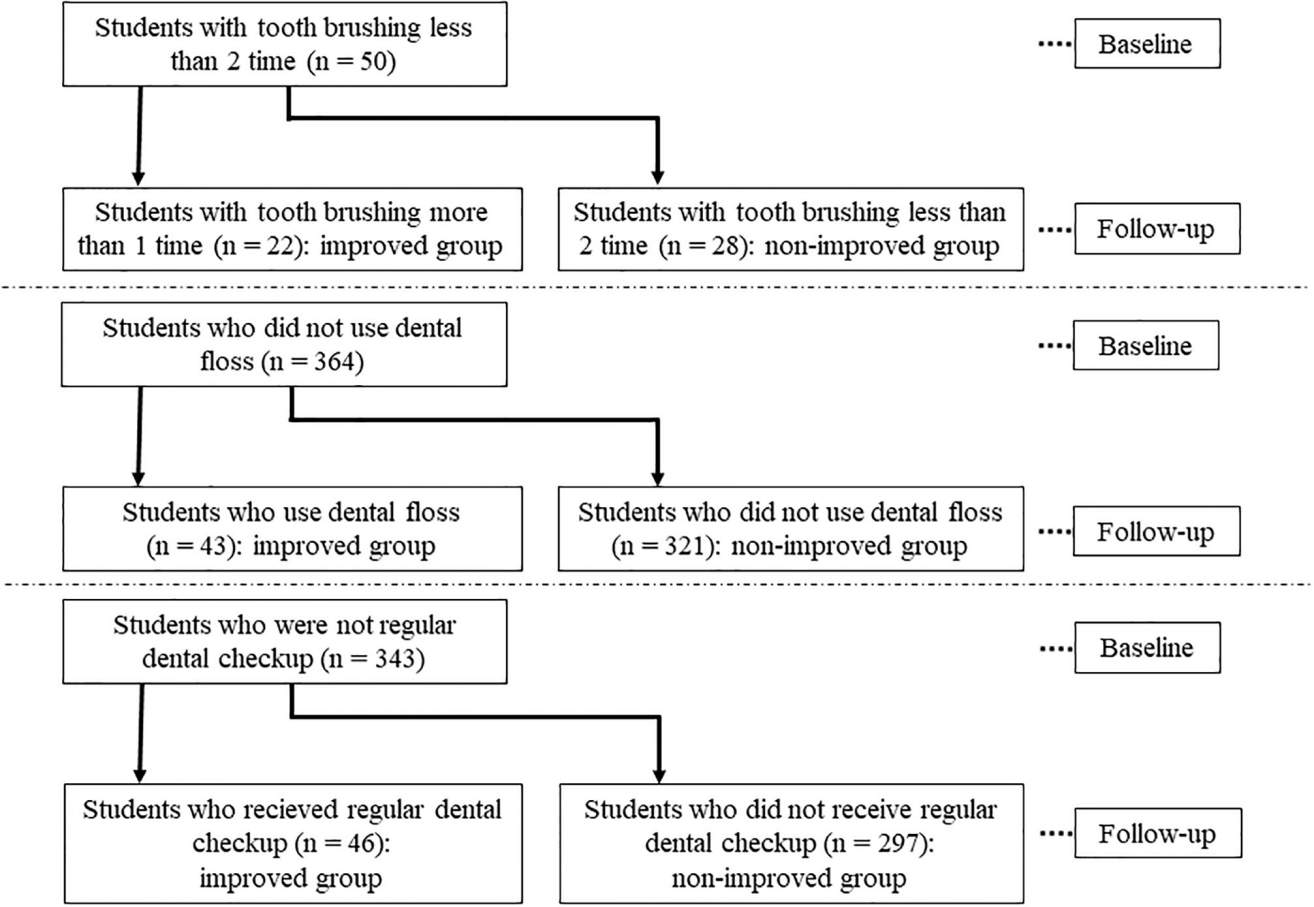
Categorization in each group. Students with poor oral hygiene behavior at baseline were categorized into improved and non-improved groups.

The percentages of students classified into the improved group for frequent tooth brushing, using dental floss, and having regular dental visits were 44.0% (22/50), 11.8% (43/364), and 15.5% (46/343), respectively (Fig 2).

### Association between oral hygiene behavior and other parameters

The students with a higher frequency of daily tooth brushing (improved group) had more oral hygiene knowledge about the “8020 movement” at both baseline (*p* = 0.029) and follow-up (*p* = 0.029) than the students with a lower frequency of daily tooth brushing (non-improved group) (Table 1). Those who started using dental floss (improved group) had significantly more oral hygiene knowledge in terms of dental plaque (*p* = 0.005) and the topical application of fluoride (*p* = 0.002) at baseline, and of calculus (*p* = 0.008) and dental floss (*p* = 0.001) at follow-up; in addition, at follow-up, dental clinics (*p* < 0.001) were found to be the source of these students’ oral hygiene knowledge (Table 2). Those who had regular dental visits (improved group) had significantly more dental knowledge in terms of the 8020 movement (*p* = 0.006) at baseline, and of calculus (*p* = 0.015) and the topical application of fluoride (*p* = 0.010) or fissure sealant (*p* = 0.007) at follow-up; in addition, family (*p* = 0.032) was found to be the source of these students’ oral hygiene knowledge at baseline, whereas television (*p* = 0.010) and dental clinics (*p* < 0.001) were found to be the sources of knowledge at follow-up (Table 3).

**Table 1:** Associations between frequency of tooth brushing per day, oral hygiene knowledge, and source of oral hygiene knowledge at baseline and follow-up.

Parameters			Improved group	Non-improved group	*p*-value^†^
			n = 22	n = 28	
Sex	Male		15 (68.2)*	22 (78.6)	0.406
Baseline					
Age			18.4 (1.1)	18.3 (0.5)	0.714
Oral hygiene knowledge	Dental plaque	Yes	5 (22.7)	6 (21.4)	1.000
	Calculus	Yes	7 (31.8)	3 (10.7)	0.084
	Periodontal disease	Yes	5 (22.7)	6 (21.4)	1.000
	8020 movement	Yes	1 (4.5)	9 (32.1)	0.029
	Temporomandibular disorder	Yes	2 (9.1)	3 (10.7)	1.000
	Dental floss	Yes	2 (9.1)	3 (10.7)	1.000
	Topical application of fluoride	Yes	0 (0.0)	1 (3.6)	1.000
	Fluoride-containing mouthwash	Yes	0 (0.0)	0 (0.0)	-
	Fissure sealant	Yes	0 (0.0)	1 (3.6)	1.000
Source of oral hygiene knowledge	Internet	Yes	3 (13.6)	4 (14.3)	1.000
	Television	Yes	7 (31.8)	11 (39.3)	0.585
	Dental clinic	Yes	11 (50.0)	19 (67.9)	0.201
	Family	Yes	5 (22.7)	8 (28.6)	0.640
	School	Yes	7 (31.8)	10 (35.7)	0.773
Follow-up					
Oral hygiene knowledge	Dental plaque	Yes	7 (31.8)	10 (35.7)	0.773
	Calculus	Yes	8 (36.4)	10 (35.7)	0.962
	Periodontal disease	Yes	2 (9.1)	6 (21.4)	0.439
	8020 movement	Yes	2 (9.1)	10 (35.7)	0.029
	Temporomandibular disorder	Yes	2 (9.1)	4 (14.3)	0.683
	Dental floss	Yes	3 (13.6)	3 (10.7)	1.000
	Topical application of fluoride	Yes	3 (13.6)	3 (10.7)	1.000
	Fluoride-containing mouthwash	Yes	2 (9.1)	1 (3.6)	0.576
	Fissure sealant	Yes	2 (9.1)	2 (7.1)	1.000
Source of oral hygiene knowledge	Internet	Yes	8 (36.4)	14 (50.0)	0.335
	Television	Yes	8 (36.4)	6 (21.4)	0.243
	Dental clinic	Yes	8 (36.4)	6 (21.4)	0.243
	Family	Yes	1 (4.5)	6 (21.4)	0.117
	School	Yes	0 (0.0)	3 (10.7)	0.246

*Number (%) and mean (standard deviation),

^†^Fisher’s exact test, chi-square test or paired *t* test

**Table 2:** Associations between the use of dental floss, oral hygiene knowledge, and source of oral hygiene knowledge at baseline and follow-up.

Parameters			Improved group	Non-improved group	*p*-value^†^
			n = 43	n = 321	
Sex	Male		19 (44.2)*	190 (59.2)	0.062
Baseline					
Age			18.3 (0.5)	18.2 (0.5)	0.539
Oral hygiene knowledge	Dental plaque	Yes	23 (53.5)	102 (31.8)	0.005
	Calculus	Yes	14 (32.6)	100 (31.2)	0.852
	Periodontal disease	Yes	8 (18.6)	69 (21.5)	0.663
	8020 movement	Yes	15 (34.9)	71 (22.1)	0.064
	Temporomandibular disorder	Yes	7 (16.3)	38 (11.8)	0.406
	Dental floss	Yes	2 (4.7)	22 (6.9)	0.753
	Topical application of fluoride	Yes	5 (11.6)	4 (1.2)	0.002
	Fluoride-containing mouthwash	Yes	0 (0.0)	1 (0.3)	1.000
	Fissure sealant	Yes	0 (0.0)	5 (1.6)	1.000
Source of oral hygiene knowledge	Internet	Yes	8 (18.6)	50 (15.6)	0.610
	Television	yes	12 (27.9)	98 (30.5)	0.725
	Dental clinic	Yes	27 (62.8)	169 (52.6)	0.210
	Family	Yes	7 (16.3)	75 (23.4)	0.296
	School	Yes	15 (34.9)	138 (43.0)	0.312
Follow-up					
Oral hygiene knowledge	Dental plaque	Yes	18 (41.9)	89 (27.7)	0.056
	Calculus	Yes	23 (53.5)	106 (33.0)	0.008
	Periodontal disease	Yes	13 (30.2)	79 (24.6)	0.426
	8020 movement	Yes	12 (27.9)	66 (20.6)	0.270
	Temporomandibular disorder	Yes	8 (18.6)	62 (19.3)	0.912
	Dental floss	Yes	10 (23.3)	21 (6.5)	0.001
	Topical application of fluoride	Yes	4 (9.3)	11 (3.4)	0.087
	Fluoride-containing mouthwash	Yes	3 (7.0)	9 (2.8)	0.158
	Fissure sealant	Yes	2 (4.7)	3 (0.9)	0.108
Source of oral hygiene knowledge	Internet	Yes	14 (32.6)	113 (35.2)	0.733
	Television	Yes	12 (27.9)	85 (26.5)	0.842
	Dental clinic	Yes	24 (55.8)	84 (26.2)	<0.001
	Family	Yes	1 (2.3)	28 (8.7)	0.228
	School	Yes	4 (9.3)	50 (15.6)	0.277

*Number (%) and mean (standard deviation),

^†^Fisher’s exact test, chi-square test or paired *t* test

**Table 3:** Associations between receiving regular dental checkups, oral hygiene knowledge, and the source of that knowledge at baseline and follow-up.

Parameters			Improved group	Non-improved group	*p*-value^†^
			n = 46	n = 297	
Sex	Male		21 (45.7)*	172 (57.9)	0.119
Baseline					
Age			18.4 (0.5)	18.2 (0.6)	0.035
Oral hygiene knowledge	Dental plaque	Yes	17 (37.0)	104 (35.0)	0.798
	Calculus	Yes	14 (30.4)	91 (30.6)	0.978
	Periodontal disease	Yes	6 (13.0)	72 (24.2)	0.092
	8020 movement	Yes	17 (37.0)	57 (19.2)	0.006
	Temporomandibular disorder	Yes	7 (15.2)	37 (12.5)	0.603
	Dental floss	Yes	2 (4.3)	24 (8.1)	0.552
	Topical application of fluoride	Yes	1 (2.2)	10 (3.4)	1.000
	Fluoride-containing mouthwash	Yes	0 (0.0)	1 (0.3)	1.000
	Fissure sealant	Yes	1 (2.2)	2 (0.7)	0.352
Source of oral hygiene knowledge	Internet	Yes	7 (15.2)	54 (18.2)	0.625
	Television	Yes	13 (28.3)	95 (32.0)	0.613
	Dental clinic	Yes	25 (54.3)	149 (50.2)	0.598
	Family	Yes	5 (10.9)	75 (25.3)	0.032
	School	Yes	15 (32.6)	130 (43.8)	0.154
Follow-up					
Oral hygiene knowledge	Dental plaque	Yes	20 (43.5)	98 (33.0)	0.164
	Calculus	Yes	25 (54.3)	106 (35.7)	0.015
	Periodontal disease	Yes	16 (34.8)	79 (26.6)	0.248
	8020 movement	Yes	13 (28.3)	58 (19.5)	0.174
	Temporomandibular disorder	Yes	10 (21.7)	60 (20.2)	0.810
	Dental floss	Yes	6 (13.0)	29 (9.8)	0.442
	Topical application of fluoride	Yes	7 (15.2)	13 (4.4)	0.010
	Fluoride-containing mouthwash	Yes	2 (4.3)	12 (4.0)	1.000
	Fissure sealant	Yes	4 (8.7)	3 (1.0)	0.007
Source of oral hygiene knowledge	Internet	Yes	12 (26.1)	118 (39.7)	0.076
	Television	Yes	5 (10.9)	86 (29.0)	0.010
	Dental clinic	Yes	34 (73.9)	67 (22.6)	<0.001
	Family	Yes	2 (4.3)	25 (8.4)	0.555
	School	Yes	5 (10.9)	47 (15.8)	0.383

*Number (%) and mean (standard deviation),

^†^Fisher’s exact test, chi-square test or paired *t* test

Multiple logistic regression analysis showed that improved change in the frequency of dental flossing was significantly associated with oral hygiene knowledge in terms of dental plaque (*p* = 0.009) and periodontal disease (*p* = 0.039) at baseline and of dental floss (*p* = 0.003) at follow up, and when television (*p* = 0.037) and dental clinics (*p* < 0.001) were found to be the source of oral hygiene knowledge at follow-up (Table 4). Further, improved change in the frequency of regular dental visits was significantly associated with age (*p* = 0.001) and oral hygiene knowledge in terms of the 8020 movement (*p* = 0.001) at baseline and of calculus (*p* = 0.043) at follow-up, and when family (*p* = 0.028) and dental clinics (*p* < 0.001) were found to be the sources of oral hygiene knowledge at baseline and follow-up, respectively. On the other hand, no significant associations were identified between the improved frequency of daily tooth brushing and other parameters.

**Table 4:** Adjusted ORs and 95% CIs for use of dental floss or regular dental visits in the logistic regression analysis.

Parameters		Use of dental floss (n = 364)				
				OR	95% Cl	*p*-value
Baseline	Oral hygiene knowledge	Dental plaque	No	1		
			Yes	2.743	1.292–5.826	0.009
		Periodontal disease	No	1		
			Yes	0.349	0.128–0.948	0.039
Follow-up	Oral hygiene knowledge	Dental floss	No	1		
			Yes	4.383	1.670–11.508	0.003
	Source of oral hygiene knowledge	Television	No	1		
			Yes	2.533	1.057–6.074	0.037
		Dental clinic	No	1		
			Yes	4.11	1.871–9.029	<0.001
Parameters		Regular dental visits (n = 343)				
				OR	95% Cl	*p*-value
Age				0.382	0.221–0.659	0.001
Baseline	Oral hygiene knowledge	8020 movement	No	1		
			Yes	4.166	1.763–9.843	0.001
	Source of oral hygiene knowledge	Family	No	1		
			Yes	0.295	0.100–0.875	0.028
Follow-up	Oral hygiene knowledge	Calculus	No	1		
			Yes	2.172	1.024–4.604	0.043
	Source of oral hygiene knowledge	Dental clinic	No	1		
			Yes	13.626	5.971–31.095	<0.001

Independent variables: sex, age, oral hygiene knowledge at baseline and follow-up, and the sources of that knowledge at baseline and follow-up.

Backward elimination method was used to select the final model.

### Relationship between oral hygiene behavior and oral condition

Among the 50 students with infrequent tooth brushing, the 364 who did not use dental floss, and the 343 who did not receive regular dental checkups, %BOP (*p* = 0.011, *p* = 0.001, *p* < 0.001, respectively), PPD (*p* < 0.001, *p* = 0.004, *p* = 0.001, respectively), and OHI-S scores (*p* < 0.001, *p* < 0.001, *p* < 0.001, respectively) were significantly higher at follow-up than at baseline (paired *t* test, chi-squared test, Wilcoxon signed-rank test) (S3 Table).

A significant increase in OHI-S scores was seen in the non-improved group compared with the improved group regarding the use of dental floss (chi-squared test; *p* < 0.001) (S4 Table). Significant increases in %BOP and OHI-S scores were also seen in the non-improved group regarding frequent dental visits (chi-squared test; *p* = 0.022, *p* = 0.030, respectively) (S4 Table). On the other hand, no significant differences in oral condition were observed between the improved and non-improved groups regarding daily tooth brushing frequency (%BOP: *p* = 0.217, PPD: *p* = 0.615, OHI-S: *p* = 0.907).

## Discussion

Based on the results of our previous cross-sectional study [11], we conducted a prospective cohort study focused on the relationship between oral hygiene knowledge, the source of that knowledge, and oral hygiene behavior. We found a significant association between improved oral hygiene behavior, in terms of the use of dental floss and having regular dental visits, and having dental clinics as the source of oral hygiene knowledge in a group of Japanese university students. As the result, it was suggested that obtaining knowledge from dental clinics could improve oral health behavior. To our knowledge, the present prospective cohort study is the first to report this longitudinal association.

In the two improved groups (use of dental floss and having regular dental visits), dental clinics were the most common source of oral hygiene knowledge (about 60%) during university life at both baseline and follow-up. Similar to the present findings, previous studies have reported that > 60% of adults obtain their oral hygiene knowledge from dentists or dental clinics [26, 27]. On the other hand, in the non-improved groups that did not use dental floss or receive regular dental checkups, the Internet was the most common source of dental knowledge. Owing to the rapid spread of the Internet in recent years, young adults have unlimited chances to obtain information on oral hygiene. However, such information from online sources can vary greatly depending on the digital literacy of the individual [28], and inaccurate information is sometimes obtained [29]. Therefore, oral hygiene knowledge obtained from Internet may not necessarily contribute to improved oral hygiene behavior, even among young adults.

Some researchers have reported on the relationship between oral hygiene knowledge and oral hygiene behavior. Muralidharan et al., reported that Indian students having oral hygiene knowledge of topical fluorides was significantly associated with good oral hygiene behavior [14]. Yao et al., reported that dental students in China had better oral hygiene behavior than medical students [15]. Márquez-Arrico et al., reported that use of dental floss was associated with good dental knowledge, however, frequency of tooth brushing did not present significant associations with levels of oral health knowledge in Spanish adult population [30]. Although it cannot be concluded, there is a general agreement that good dental knowledge and good oral health behavior are generally correlated.

In this study, having the knowledge of the 8020 movement was significantly associated with improved regular dental visit. The 8020 movement is a Japanese social campaign aiming to retain 20 or more of one’s own teeth at the age of 80. The participants who had the knowledge of the 8020 movement may be willing to do regular dental visit to retain 20 or more of one’s own teeth at the age of 80. The previous study also shows that the dental knowledge including the 8020 movement is significantly associated with regular dental visit [10] and supports our study.

No significant association was found between the frequency of tooth brushing and the source of oral hygiene knowledge; this finding supports that of our previous study [11].

On the other hand, it has been reported that the frequency of tooth brushing was associated with sex [31] or knowledge of topical fluoride [14], these results contradicted the present study. However, we should pay attention to the number of participants. Many students (86.3%) regularly brushed their teeth more than twice a day, so there might not have been much room for improvement, thereby creating a type of ceiling effect. Further, in this study the minimum sample size was 187. In the case of improved tooth brushing, the number of analyzed participants was less than the required number. The results of this analysis need to be interpreted with caution.

Not using dental floss was associated with lower OHI-S scores, and not receiving regular dental checkups was associated with lower OHI-S scores and %BOP (S3 Table); these findings suggest that the students with poor oral hygiene behavior also had poor oral hygiene and/or periodontal status, which supports the findings of previous studies [6, 10, 11]. Therefore, acquiring oral hygiene knowledge from dental clinics may be effective for improving oral hygiene behavior, thereby contributing to the achievement and maintenance of good periodontal status.

In the present study, the prevalences of frequent tooth brushing (≥ twice/day), dental floss use, and regular dental visits among all participants at baseline were 86.3%, 13.3%, and 17.0%, respectively. According to the Japanese Survey of Dental Diseases (2016) (https://www.mhlw.go.jp/toukei/list/62-28.html), the prevalences of frequent tooth brushing (≥ twice/day) and dental floss use among young people (age 20–24 years) were 77.0% and 20.4%, respectively; these data were similar to those in the present study. On the other hand, according to the Japanese National Health and Nutrition Survey (2016) (https://www.mhlw.go.jp/stf/houdou/0000177189.html), the prevalence of receiving regular dental checkups among young people aged 20–29 years was 43.3%. Okayama University students may have a lower rate of regular dental checkups. Therefore, the participants in this study should be carefully evaluated.

This study did have some limitations. First, other possible confounders, such as lifestyle, stress and education levels, and socioeconomic status, were not included in the analysis. Second, given the low follow-up rate (16.1–18.5% of all eligible students), there is a possibility of a selection bias. Third, we did not assess the effects of other variables that might affect oral hygiene behavior, such as the frequency of obtaining information from the given source, the interactions between knowledge and knowledge sources, the frequency of dental floss use, the recall interval for dental checkups, and dental visit patterns, or the relation between oral health status and dental visit patterns. Forth, we could not test participants’ knowledge on preventive measures actually because we could only include the questionnaire during the routine oral health examination. Therefore, we could not investigate whether participants have the correct knowledge. Finally, because all participants were recruited from the same university in Japan, caution is needed in extrapolating these findings to the general population of younger people.

## Conclusion

The results of the present 3-year cohort study suggest that having oral hygiene knowledge and obtaining knowledge from dental clinics among Japanese university students could improve oral health behavior in terms of the use of dental floss and regular dental visits. Acquiring oral hygiene knowledge from dental clinics may be effective for contributing to the achievement and maintenance of good periodontal status.

## Supporting information

S1 Table Questionnaire list in English. (PDF)

S2 Table Questionnaire list in Japanese. (PDF)

S3 Table Differences in periodontal status and oral hygiene at baseline and follow-up. (PDF)

S4 Table Differences in worsened periodontal status and oral hygiene between the improved and the non-improved groups. (PDF)
