# Supplementary material for: Relationship between oral hygiene knowledge, source of oral hygiene knowledge and oral hygiene behavior in Japanese university students: A prospective cohort study
Source: PLoS One. 2020 Jul 23;15(7):e0236259. doi: 10.1371/journal.pone.0236259 (PMC7377407; doi:10.1371/journal.pone.0236259)
Supplement: S1 Table — (PDF) [file pone.0236259.s001.pdf]

S1 Table. Questionnaire list in English.

Questionnaire	Answer
Which dental terms can you explain? (multiple selection)	dental plaque, calculus, periodontal disease, 8020 movement, temporomandibular disorder, dental floss, topical application of fluoride, fluoride-containing mouthwash, fissure sealant
Where have you acquired most of their oral hygiene knowledge? (multiple selection)	internet, television, dental clinics, family, school
How many times do you brush your teeth in a day?	≥ twice/≤ once
Do you use dental floss?	yes/ no
Have you received regular dental checkups during the past year?	yes/ no
