# Supplementary material for: Relationship between oral hygiene knowledge, source of oral hygiene knowledge and oral hygiene behavior in Japanese university students: A prospective cohort study
Source: PLoS One. 2020 Jul 23;15(7):e0236259. doi: 10.1371/journal.pone.0236259 (PMC7377407; doi:10.1371/journal.pone.0236259)
Supplement: S2 Table — (PDF) [file pone.0236259.s002.pdf]

S2 Table. Questionnaire list in Japanese.

質問	回答
歯科に関する用語の中で説明できるものはありますか？（複数選択可）	歯垢／歯石／歯周病／8020 運動／顎関節症／デンタルフロス／フッ化物歯面塗布／フッ化物洗口／シーラント
口腔に関する知識をどこから得ましたか？（複数選択可）	インターネット／テレビ／歯科医院／家族／学校
1 日に何回歯を磨きますか？	2 回以上／1 回以下
デンタルフロスを使用していますか？	はい／いいえ
過去 1 年間に歯科医院へ定期受診に行きましたか？	はい／いいえ
