# Supplementary material for: Relationship between oral hygiene knowledge, source of oral hygiene knowledge and oral hygiene behavior in Japanese university students: A prospective cohort study
Source: PLoS One. 2020 Jul 23;15(7):e0236259. doi: 10.1371/journal.pone.0236259 (PMC7377407; doi:10.1371/journal.pone.0236259)
Supplement: S3 Table — (PDF) [file pone.0236259.s003.pdf]

S3 Table. Differences in periodontal status and oral hygiene between the baseline and the follow-up.

Parameters		baseline		after 3 years		*p*-value§
Daily frequency of tooth brushing (≤ 1 time) at baseline (n = 50)						
%BOP		31.0	± 27.9*	44.0	± 22.5	0.011
Presence of PPD ≧4 mm	-	45	(90.0)†	19	(38.0)	<0.001
	+	5	(10.0)	31	(62.0)	
OHI-S		0.4	(0, 1.0) ‡	1.2	(0, 1.8)	<0.001
Non dental floss users at baseline (n = 364)						
%BOP		32.2	± 27.6	40.05	± 25.0	0.001
Presence of PPD ≧4 mm	-	307	(84.3)	166	(45.6)	0.004
	+	57	(15.7)	198	(54.4)	
OHI-S		0.3	(0, 0.7)	0.7	(0, 1.5)	<0.001
Non regular dental visitors at baseline (n = 343)						
%BOP		31.3	± 27.8	39.8	± 25.0	<0.001
Presence of PPD ≧4 mm	-	293	(85.4)	162	(47.2)	0.001
	+	50	(14.6)	181	(52.8)	
OHI-S		0.3	(0, 0.7)	0.7	(0, 1.5)	<0.001

* Mean ± standard deviation, † Number (%), ‡ Median (25 percentiles, 75 percentiles), § Paired t-test, McNemar–Bowker test or Wilcoxon signed-rank test.
