# Supplementary material for: Relationship between oral hygiene knowledge, source of oral hygiene knowledge and oral hygiene behavior in Japanese university students: A prospective cohort study
Source: PLoS One. 2020 Jul 23;15(7):e0236259. doi: 10.1371/journal.pone.0236259 (PMC7377407; doi:10.1371/journal.pone.0236259)
Supplement: S4 Table — (PDF) [file pone.0236259.s004.pdf]

S4 Table. Differences in worsened periodontal status and oral hygiene between the improved and the non-improved groups.

Parameters	Improved group		Non-improved group		*p*-value†
Daily frequency of tooth brushing (≤ 1 time) at baseline (n = 50)	n=22		n=28		
Worsened %BOP	12	(54.5)*	20	(62.5)	0.217
Worsened PPD	11	(50.0)	16	(57.1)	0.615
Worsened OHI-S	13	(59.1)	17	(60.7)	0.907
Non dental floss users at baseline (n = 364)	n=43		n=321		
Worsened %BOP	25	(56.1)	175	(54.5)	0.654
Worsened PPD	23	(53.5)	134	(41.7)	0.144
Worsened OHI-S	9	(20.9)	163	(50.8)	<0.001
Non regular dental visitors at baseline (n = 343)	n=46		n=297		
Worsened %BOP	19	(41.3)	176	(59.3)	0.022
Worsened PPD	18	(39.1)	126	(42.4)	0.674
Worsened OHI-S	18	(39.1)	167	(56.2)	0.030

* Number (%), † Chi-square test
